# Effects of a digital visual art learning intervention in healthy older adults: a pilot randomized controlled trial

**DOI:** 10.3389/fragi.2025.1635789

**Published:** 2025-09-04

**Authors:** Akari Uno, Ryan Browne, Takamitsu Shinada, Michio Takahashi, Keishi Soga, Yegang Du, Fumihira Abiko, Yasuyuki Taki

**Affiliations:** ^1^ Smart Aging Research Center, Tohoku University, Sendai, Japan; ^2^ Department of Psychology, Faculty of Humanities, Saitama Gakuen University, Kawaguchi, Japan; ^3^ Division of Teacher Training for the Arts, Physical Education and Lifestyle Education, Miyagi University of Education, Sendai, Japan; ^4^ Department of Aging Research and Geriatric Medicine, Institute of Development, Aging and Cancer, Tohoku University, Sendai, Japan

**Keywords:** digital visual art, digital arts learning, cognitive impairment, aging population, randomized controlled trial

## Abstract

**Introduction:**

This study aimed to examine the effects of a digital visual art learning intervention on the cognitive and psychological functions of healthy older adults with no prior experience in art.

**Methods:**

An open-label pilot randomized controlled trial was conducted, with participants (mean age = 69.43 ± 2.70 years) aged 65 to 74 assigned to either an intervention (n = 37) or control (n = 35) group. Cognitive and psychological functions were assessed before and after 20 sessions of digital visual art learning. In each session, participants mainly worked on lectures and prepared tasks related to basic themes in art. In the final stage, each participant engaged in individual creative activities and aimed to complete their works.

**Results:**

In the Frontal Assessment Battery, which evaluates frontal lobe function, a significant trend was observed in the interaction between group and time (p = 0.062), and although an improvement trend was observed in the intervention group, the change did not reach statistical significance.

**Conclusion:**

These findings suggest that digital art could potentially enhance executive function in older adults, providing important insights into its applicability within this population.

**Clinical trial registration:**

https://center6.umin.ac.jp/cgi-open-bin/ctr_e/ctr_view.cgi?recptno=R000058589, identifer UMIN000051427

## 1 Introduction

The world’s population of adults aged 65 years and older has tripled in approximately 40 years from 258 million in 1980 to 771 million in 2022 and is expected to increase by a further 17% by 2050 (United Nations Department of Economic and Social AffairsPopulation Division, 2022). Under these circumstances, ensuring that older adults maintain healthy lifestyles is an important issue both for individuals and society as a whole. For older adults to live healthy lives, they should have access to learning opportunities, regardless of their age (Hardy et al., 2017; Schoultz et al., 2020). The World Health Organization (World Health Organization, 2015) has recommended lifelong learning and growth for all, regardless of individual ability level, by emphasizing the importance of creating accessible opportunities. Furthermore, society—with increased expectations for the potential of older adults—has undergone a shift in attitudes toward old age, now thought of as a “period of opportunity and wellbeing, with retention, or development, of the psychological resources to cope with life’s challenges” (Bowling, 2007). This highlights the need to create an environment in which older adults can continue to learn new things and assume new challenges.

Among various learning opportunities, participation in artistic activities has been shown to contribute to improvements in cognitive function, mental health, physical functioning, and quality of life among older adults, thereby promoting healthy aging (Groot et al., 2021; Noice et al., 2014; Tymoszuk et al., 2020). Research using large-scale data from the Wisconsin Longitudinal Study reported that, even after adjusting for confounding factors, engaging in participatory arts for up to 1 hour weekly and receptive arts for up to 3 hours weekly enhanced executive and language functions (Bone et al., 2024). Additionally, continuous participation in participatory art workshops held at the Montreal Museum of Fine Arts was shown to improve the quality of life and health status of community-dwelling older adults (Beauchet et al., 2020). These findings collectively suggest that engagement in artistic activities offers substantial benefits for the health and wellbeing of older adults.

This study focuses on digital art, a relatively new form of artistic expression. As a genre of contemporary art created using digital technologies, digital art expands the boundaries of traditional art and enhances accessibility through technological integration (Srivastava, 2019). Moreover, the use of digital tools promotes greater interaction with science, technology, media, and computers, thereby stimulating aesthetic exploration (Özdemir, 2022). The scope of digital art is broad, but this study focuses specifically on digital visual art characterized by drawing. While digital art encompasses multimedia works and installations, the digital visual art addressed in this study is defined as “activities that involve drawing, painting, photo editing, and other visual expressions using digital devices such as tablet devices and pen tablets.” Digital visual art offers several advantages over traditional analog art, including expanded expressive possibilities, high portability, and ease of operation—specifically, the ability for users to complete tasks with just the touch of a finger (Darewych et al., 2015). Analog art involves the burden of preparing and cleaning tools and materials, compensating line instability due to hand tremors and pen pressure adjustments, and dealing with the fear of the irreversibility of mistakes during the process of creating artwork; the characteristics of digital art, however, can overcome these barriers, providing a more accessible gateway to art activities for older adults. Furthermore, learning multiple real-world skills, including drawing, can enhance cognitive function in older adults (Leanos et al., 2023). Therefore, environments where both artistic and digital skills can be acquired simultaneously through digital visual art are expected to offer significant advantages for older adults. However, it has also been reported that a certain proportion of older adults experience anxiety or resistance when using digital technologies (Di Giacomo et al., 2019; Kim et al., 2023). Thus, it is essential to examine whether digital visual art can serve as a content area that is easily adaptable and accessible for older populations.

Furthermore, compared to traditional art, there is an overwhelming lack of research examining the effects of digital visual art, a topic that necessitates more detailed consideration when aiming for broad application among the older population. Intervention studies on older adults using traditional visual arts—concerning which much knowledge has been accumulated—have reported improvements in various cognitive domains, including global cognitive function (Cetinkaya et al., 2019; Ciasca et al., 2018; Zhao et al., 2018), memory (Lee et al., 2019; Mahendran et al., 2018; Noice et al., 2004), attention (Lee et al., 2019; Mahendran et al., 2018), executive function (Brown et al., 2021; although this intervention primarily involved conceptual art, elements of visual art were included), and cognitive flexibility (Brown et al., 2021). Drawing activities have been suggested to be associated with various domains of cognitive functioning and to promote neuroplasticity (Bolwerk et al., 2014). Among these domains, executive function, which is closely linked to frontal lobe activity, appears to be particularly relevant. Executive function refers to complex cognitive processes that require the integration and coordination of multiple sub-processes to achieve goal-directed behavior (Elliott, 2003). Previous studies investigating the relationship between creative drawing and brain activity have reported increased activation of neural networks associated with executive functions (Bolwerk et al., 2014; Chamberlain et al., 2014; De Pisapia et al., 2016; Raimo et al., 2021). Furthermore, decline in executive functioning is considered a prominent feature of cognitive aging, with significantly lower scores observed among older adults compared to younger individuals (Idowu and Szameitat, 2023). Given this background, creative drawing activities may serve as a promising approach to support and enhance executive functioning in older adults. In terms of psychological outcomes, interventions using traditional visual arts have been shown to reduce depressive symptoms (Ciasca et al., 2018; Hattori et al., 2011; Kang et al., 2010; Kongkasuwan et al., 2016; Pongan et al., 2017) and anxiety (Ciasca et al., 2018). Moreover, a review by McQuade and O’Sullivan (2023) revealed that engagement with visual arts may also contribute to improvements in social factors, such as reduced feelings of loneliness and enhanced sense of community and social connectedness.

In contrast, only a limited number of studies have explored the use of digital art in practice (Choe, 2014; Kaimal et al., 2019; Kim and Chung, 2023; Darewych et al., 2015). While none of these studies specifically targeted older adults, and all four adopted qualitative approaches, they not only highlighted the potential of digital art to enhance mood and facilitate the exploration of creative play in ways that may not be possible in the physical world (Kaimal et al., 2019) but also provided opportunities for enjoyable artistic engagement and vivid expression of positive emotions (Kim and Chung, 2023). Choe (2014) investigated the advantages and disadvantages of using iPads for digital art in art therapy through interviews with experts. While disadvantages include potential technical difficulties, the addictive nature of internet-connected devices, the limited size of the screen (working canvas) compared to traditional media, and a lack of messiness; advantages include being able to easily edit (redo and undo) work, the portability of using a handheld device, the ability to create multiple outcomes of an image, and interactive features. A recent comprehensive review on Digital Creative Art Interventions (DCAIs) targeting older adults identifies DCAIs as an innovative approach to arts-based interventions utilizing digital technologies. The review provides an overview of previous studies in this field, indicating that DCAIs have been implemented through various modalities, including music, dance, museum experiences, photo collages, theater, visual art interactions, and multimodal artistic activities (Du et al., 2025). Moreover, it highlights the substantial potential of DCAIs to serve as novel approaches for promoting the physical and mental health of older adults. However, within this review, only one study addressing visual art was identified, and notably, the intervention did not involve participants in active artistic creation such as drawing or painting. Instead, it focused on full-body engagement with traditional visual art content in an interactive manner (Camurri et al., 2024). This finding underscores the significant lack of empirical evidence regarding visual art interventions utilizing digital technologies specifically designed for older populations. As previously discussed, digital visual art holds several unique features that may enhance, complement, or even yield distinct effects compared to traditional art-based interventions. Given this, the current paucity of research addressing the potential cognitive and psychological benefits of digital visual art among older adults represents a critical gap in the literature. It is therefore essential to investigate how both digital and traditional forms of visual art impact the cognitive and psychological functioning of this population.

Thus, the purpose of this study is to examine the effects of digital visual art learning intervention on the cognitive and psychological functions in healthy older adults. We hypothesize that utilizing digital visual art learning will contribute to new approaches to extend older adults’ healthy life span and improve their quality of life.

## 2 Methods

### 2.1 Research design and procedure

This study was designed as a quantitative, longitudinal, and experimental study, employing an open-label pilot randomized controlled trial (RCT) framework. The quantitative approach was chosen to enable objective measurement of cognitive and psychological outcomes, while the longitudinal design allowed for tracking changes over a 6-month intervention period. The experimental nature of the study, specifically the use of randomized controlled trials was intended to provide evidence regarding the effectiveness of interventions involving digital visual art learning. The decision to position this research as a pilot trial was based on the novelty of the intervention and the limited number of prior empirical studies targeting healthy older adults in this context. A pilot approach was deemed appropriate to generate foundational data and assess feasibility, thereby informing the development of a future large-scale trial.

The intervention group (IG) and the control group (CG) were compared to evaluate the effects of participating in 20 digital art learning sessions on outcome measures assessed at pre- and post-intervention time points. IG participants were required to engage continuously in digital visual art activities, whereas CG participants were instructed to maintain their usual routines during the 6-month intervention period and refrain from actively participating in any art-related activities.

This study was conducted at the Institute of Development, Aging and Cancer, Tohoku University, in Sendai, Miyagi Prefecture, Japan, from October 2023 to January 2025. Participants were randomly assigned to the IG and CG using random computer-generated numbers linked to the ID of each participant. The study was an open-label pilot RCT, and both researchers and participants were aware of their assignments.

**FIGURE 1:**
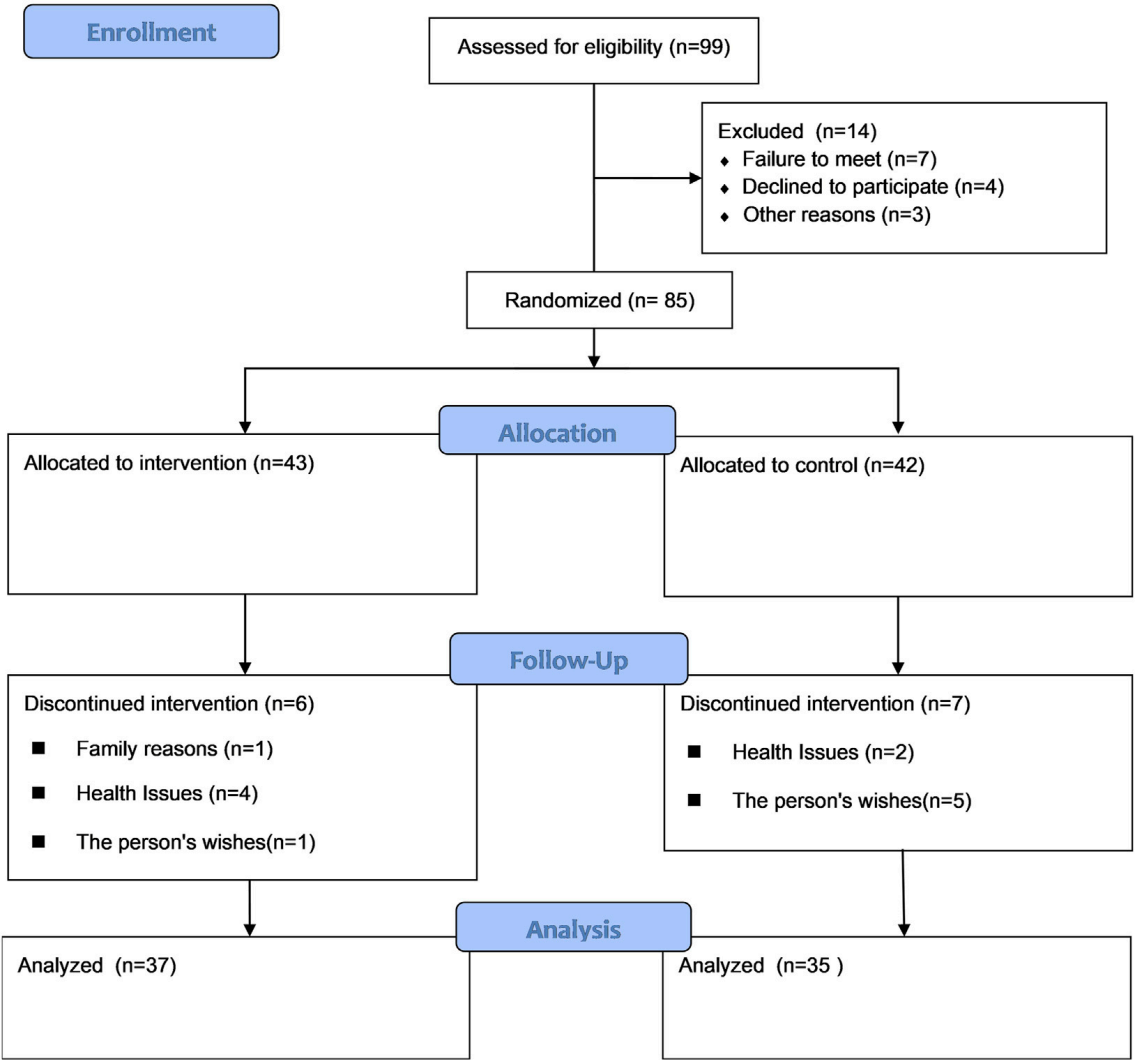
CONSORT flow diagram.

The study followed the CONSORT guidelines, as illustrated in Figure 1. Participants were recruited from community-based settings through advertisements in local information magazines. To ensure the availability of intervention resources and maintain intervention quality, it was conducted in two separate periods. In the first, participants were recruited in October 2023, followed by assessments and the intervention from November 2023 to June 2024. In the second, participants were recruited in May 2024, followed by assessments and the intervention from June 2024 to January 2025. The detailed study protocol has been described elsewhere (Uno et al., 2025).

### 2.2 Participants

The participants were healthy older adults aged 65–74 years. This age group corresponds to the “young-old” population (ages 65–74) in Japan. As health-related risks increase with advancing age (Guo et al., 2022), this age range was selected with consideration for cognitive and physical burden, as well as for the participants’ adaptability to tablet-based operations and their relatively high potential for sustained engagement in the intervention. In addition, In this study, “healthy” was defined as individuals who were generally in good health, with no history of dementia or neurological disorders, and living independently in their own homes.

The inclusion criteria were as follows: (1) males or females aged between 65 and 74 at the time of enrollment; (2) living independently at home; (3) no prior experience of painting beyond school-based art classes; and (4) willingness to provide written informed consent to participate in the study. The exclusion criteria were: (1) a history of dementia or neurological disorders; (2) severe visual or hearing impairments; (3) diagnosis of a psychiatric disorder; and (4) concurrent participation in another clinical study.

A total of 85 individuals consented to participate in the study. However, 13 participants (6 from the IG and 7 from the CG) withdrew, resulting in a dropout rate of 15.30%. Ultimately, 72 participants (37 in the IG and 35 in the CG) completed all phases of the study. Their data were included in the final analysis.

### 2.3 Assessment instruments

#### 2.3.1 Demographic variables

Participants were asked to report their age, sex, years of education, current medical history, previous medical history, medication intake, and supplement intake. Medication intake, and supplement intake were obtained as supplementary information and were not used in the analysis of this study.

#### 2.3.2 Measurement of cognitive function

The Frontal Assessment Battery (FAB) (Dubois et al., 2000) was used to measure overall frontal lobe function, including executive function. It consists of six domains: (1) conceptualization (answering categories of two or three words), (2) mental flexibility(recalling as many words as possibly that starts with a specified letter in 1 minute), (3) motor programming (imitating Luria’s rock-paper-scissors hand series), (4) sensitivity to interference (two-one-tapping task in which the participant must show the opposite response to the examiner’s alternating signals), (5) inhibitory control (go/no-go task, in which the participant must suppress a previously given response to the same stimulus), and (6) grasping behavior(the examiner extends their hand and instructs the participant not to touch it and checks for abnormal behaviors such as grasping). Each item is scored on a scale of 0–3 points. Total scores ranged from 0 to 18 points. The reliability and validity of the Japanese version have been confirmed in Nakaaki et al. (2007).

The Rey Auditory Verbal Learning Test (RAVLT) was used to assess language memory (immediate recall, delayed recall, and recognition) (Rey, 1964; Schmidt, 1996). The RAVLT is an internationally widely used neuropsychological test, and its convergent validity and test-retest reliability have been confirmed in previous studies (de Sousa Magalhães et al., 2012). Additionally, the RAVLT has been suggested to predict the progression from MCI to dementia (Moradi et al., 2017). In this study, we used the Japanese version (Wakamatsu et al., 2003) developed based on the same paradigm. After learning one list of words and working on another list of words as an interference task, participants were asked to recall the first 15 words again (immediate recall). Thirty minutes later, they were asked to replay the list of words (delayed recall) and re-recognize them. The number of correct responses was used as the score for the list study, immediate replay, delayed replay, and recognition.

The Trail Making Test (TMT) was used to assess processing speed, sequencing, cognitive flexibility, and visuomotor skills (Bowie and Harvey, 2006). Its reliability and validity have been demonstrated in previous studies (Sánchez-Cubillo et al., 2009; Wagner et al., 2011). In this study, a Japanese version of the TMT developed based on the same paradigm was used (Japan Society for Higher Brain Function, 2019). In Part A, participants were asked to connect 25 randomly scattered numbers in sequence with a line as quickly as possible. In Part B, they were asked to connect numbers and letters with alternating lines; both parts used trial time as a score, with shorter trial times indicating higher performance. The difference between in trial time Part A and Part B—ΔTMT—was used to assess cognitive flexibility-.

The Japanese version of the Cognitive Flexibility Inventory was used cognitive flexibility (Dennis and Vander Wal, 2010; Tokuyoshi and Iwasaki, 2012). It consisted of 16 items and 7 scales, with a score range of 16–112 points. The reliability and validity of the Japanese version have been confirmed by Tokuyoshi and Iwasaki (2012).

The Stroop task was used to measure inhibitory control. In this study, we used a Stroop and reverse Stroop task developed by Hakoda and Watanabe (2005). The test consisted of four tasks. In Task 1 (reverse Stroop control), participants selected a color patch corresponding to the meaning of a color word printed in black ink. In Task 2 (reverse Stroop interference), they selected the color patch matching the meaning of a color word printed in an incongruent ink color. In Task 3 (Stroop control), participants selected a color patch corresponding to the meaning of a black-ink color word. In Task 4 (Stroop interference), they focused on the ink color of an incongruent color word and selected the matching word from a list printed in black. Each trial lasted 60 s and was preceded by a practice session. In this study, we used the Stroop interference rate and the reverse Stroop interference rate as indices, referring to previous studies (Hakoda and Sasaki, 1990). This method calculated the degree of interference proportionally based on the difference in the number of correct answers between control tasks and interference tasks, while eliminating the effects of speech rate and motor ability through a matching reaction pattern (Hakoda and Sasaki, 1990). This method demonstrated high retest reliability and showed no practice effect or order effect. A lower interference rate indicated better performance. The calculation formula is as shown below:

$$\text{Stroop interference}=\left(\text{number of correct answers in the color}-\text{word task}-\text{number of correct answers}\text{ in the Stroop task}\right)/\left(\text{number of correct}\text{ answers in the color}-\text{word task}\right)\times 100$$

$$\text{Reverse Stroop interference}=\left(\text{number of correct responses in }\text{the word}-\text{color task}-\text{number of}\text{ correct responses in the reverse }\text{Stroop task}\right)/\left(\text{number of correct}\text{ responses in the word}-\text{color task}\right)\times 100$$

The Digital Cancellation Test (D-CAT) was used to measure attentional function (Hatta et al., 2023) across three administrations. In the first administration, one designated number was deleted from a random sequence of numbers, and thereafter the number to be deleted increased one by one to measure. In this study, the workload, which is the total number of retrieved numbers, was used as the measurement index, with higher workloads indicating better attentional function. The reliability and validity have been confirmed by Hatta et al. (2012).

Forward and backward Digit Span were used to measure short-term memory and working memory, respectively (Wechsler, 1987). Participants were asked to play the numbers in the order in which they were read by the examiner in the former (forward), and in the reverse order (backward) in the latter. The number of correct answers was used as the score. Standardized in Japanese and confirmed for reliability and validity (Nihon Bunka Kagakusha, 2018).

Cognitive function tests were performed in the order described above. RAVLT-delayed replay was administered after the completion of the Digit Span, ensuring that 30 min had elapsed since the RAVLT-immediate replay.

#### 2.3.3 Measurement of psychological function

To measure mental health, we used a shortened version of the Japanese version of the Geriatric Depression Scale 15 (GDS-15-J) (Sugishita et al., 2017), which requires a “yes” or “no” response to 15 items. Total scores ranged from 0 to 15 points, with higher scores indicating poorer mental health. The reliability and validity have been confirmed by Sugishita et al. (2016).

To measure life satisfaction and happiness, we used the Japanese version of the Life Satisfaction Scale (SWLS) (Oishi, 2009), which consists of 5 items and is rated on a 7-point scale. Total scores ranged from 5 to 35 points, with higher scores indicating higher life satisfaction and happiness. The reliability and validity have been confirmed by Oishi (2009).

#### 2.3.4 Measurement of drawing skills

**FIGURE 2:**
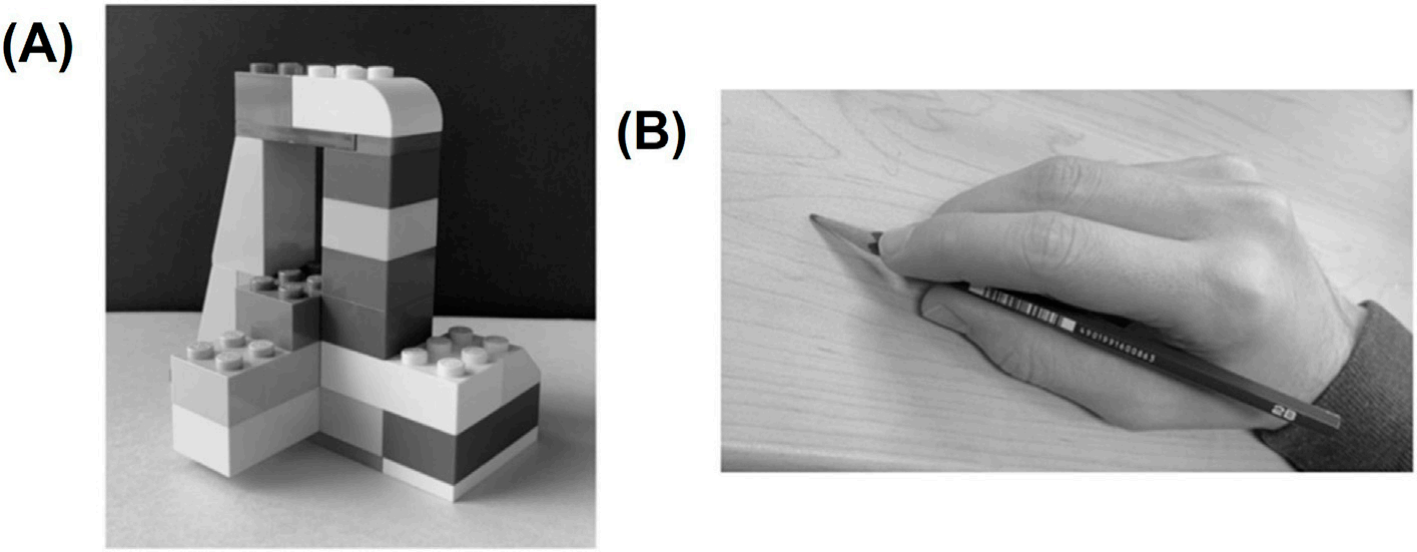
Drawing skills tasks: **(A)** Task A (block); **(B)** Task B (hand with pencil).

In IG, drawing ability tests were conducted during the 1st and 20th lessons. Task A (block) and Task B (hand with pencil) were selected based on previous studies (Chamberlain et al., 2014), and drawing tasks were performed with a digital pen tablet while the black and white photographs shown in Figure 2 were presented to participants. The scoring was developed with reference to previous studies (Rincon-Gómez et al., 2016) and was based on four aspects: line (line quality and stability), proportion (spatial perception of parts and the whole), accuracy (representation of detailed parts), and perspective (depth and relative position). Each aspect was scored from 0 to 3. Scores for Task A and Task B ranged from 0 to 24 points. Scoring was done by a university professor specializing in fine arts.

### 2.4 Health-oriented intervention protocol: digital visual art learning

There are no previous empirical studies on structured digital visual art interventions targeting healthy older adults. Since such interventions are pioneering efforts, the digital visual art learning program in this study was developed based on discussions and consensus among experts in art education and educational practice, including university professors specializing in art, and digital art practitioners. The goal of the intervention was to engage older adults—all of whom were art beginners—in learning digital visual arts. All interventions were conducted in the same location within the university facility where the required digital equipment was installed.

The IG was required to participate in a total of 20 weekly 90-min digital visual art lessons over a 6-month period. Each group was further subdivided into four to six members who attended the lessons. One or two researchers specializing in psychology or neuroscience and one or two art club students were in attendance to provide guidance and support for each session. Prior to the start of the intervention, participants received several lectures on basic digital pen tablet operation by a staff member of the digital pen tablet development company (Wacom Corporation, Saitama, Japan) to ensure that they acquired certain skills. At the beginning of each lesson, the researcher gave a lecture on the day’s theme for approximately 10 min, following which the participants were asked to work on a task based on that theme for approximately 60 min. Several types of low-to high-difficulty tasks were available, and the participants were asked to choose the one best suited to their ability level. At the end of the lesson, participants had time to reflect on the day and share their impressions of each other’s work. They were allowed to take a break at any time during the lesson, chat with other participants, and engage in a free atmosphere.

The content of the 20 lessons was divided into five steps: (1) practice with basic digital pen tablets, (2) shading in black and white, (3) color practice, (4) other important topics (such as perspective), and (5) creating a final artwork. The themes and activities of each session of the digital art learning intervention are presented in Table 1. The digital drawing device used in the lessons was a Wacom Cintiq-16 (Wacom Corporation, Saitama, Japan). Clip Studio Paint (Celsys, Tokyo, Japan) was the drawing software used.

**TABLE 1:** Contents of each session.

#	Theme	Activities	Objects	Expected outcomes
Practice with basic digital pen tablets				
#1	Explanation of the entire program Explanation of basic functions of pen tablets and software Introduction to the use of layers, drawing ability test	Trial writing, photo tracing	Become familiar with digital tools and the overall structure of the program	To understand the purpose of the program as a whole Become familiar with drawing activities To learn basic operation of equipment and software To learn the basic operation of the equipment and software To become familiar with hand/visual interaction and digital drawing through tracingetc.
#2	Manipulation exercises, shapes and shadows	Image tracing, layer duplication, and bucket functions	Understanding the basics of form in painting and learning the tools and techniques for drawing	
Practice describing in black and white				
#3	Expression of three-dimensional objects	Wire mesh writing over the image, observation drawing utilizing auxiliary lines	Better understanding of spatial structure and depth	Acquire three-dimensional expression based on observation and the ability to distinguish between light and shadow Deepening understanding of complex line drawings and visual composition, such as composition and focus Even in monochrome expressions without color, it will be possible to express three-dimensionality and spatiality by utilizing shadows and shades Improved operability of digital tools and increased concentration on drawing
#4	Line practice	Reproduce complex line shapes such as tree trunks and rock formations	Improve accuracy and variation of lines	
#5	Expression of light and dark	Gradation exercises using pencil and pastel tools	Learn the basics of gradation and light/dark expression	
#6	Review of useful tools (undo/redo, zoom, etc.)	Observation drawing of black and white photographs for assignment, shading using pastel tools	Review of techniques learned so far	
#7	Application of shading (core shadow, projected shadow)	Drawing practice using burg plates	Learn how to shade more accurately	
#8	Composition basics (3 partitioning method, focal point)	Composition with cropped images (digital collage), use of selection tools	Learn the basics of composition in painting	
Color practice				
#9	Introduction to color theory (Hue, Saturation, Lightness)	Colorizing a black-and-white photo (using the “Colorize” layer property)	Understand the relationship between light and color and learn to operate basic coloring tools	To understand the concepts of hue, saturation, and lightness, and the basics of color psychology, such as warm and cool colors To be able to blend colors and process boundaries according to purpose using palettes and brush settings To be able to improve expression and creativity through color and to explore personal style
#10	Concept of warm and cold colors	Sketching exercise using a limited palette	Understand the characteristics of warm and cold colors	
#11	Edge processing basics (hard and soft edges) Color Blending Techniques	Parallel copying exercises with full paletteParallel copying exercises with full palette	Learn to operate more advanced tools to enable detailed expression	
#12	Understanding of brush types and important settings (opacity, etc.)	Explore your own style by experimenting with different brushes	Try out various brush types to find the one that best suits your image	
Other important topics				
#13	Perspective basics	Exercises on the subject of landscape painting	Learn basic perspective	To be able to draw the spatial composition of landscapes and still lifes based on an understanding of perspective To be able to clarify the theme you want to express and select the appropriate composition and viewpoint Develop compositional skills and judgment by comparing and considering multiple ideas through thumbnail sketches
#14	Application of perspective techniques	Exercises on the subject of still life painting	Learn more advanced perspective and how to place viewpoints in paintings	
#15	Consideration of ideas using thumbnail sketches	Create multiple versions of the painting and study the overall composition of the painting	Learn how to develop ideas, which is important in creating artwork	
Creating a final artwork				
#16	Review of key lessons and final project preparation	Selection of objects and photographs to be used	Reflection on previous studies	Integrate what you have learned so far and reflect it in a single finished work Not only will you develop creativity and expressiveness, but you will also cultivate independence, concentration, and perseverance Through the creation of a finished work, you will gain a sense of accomplishment and self-affirmation
#17–19	Final project production	While we encourage the completion of one work on a favorite subject of the participants, multiple creations are also acceptable	Through the creation of a final project, participants will put into practice the knowledge and skills they have learned in the program	
#20	Final project production, drawing ability inspection	Completion of final artwork	The goal is to complete the final work	

To prevent interruptions to study participation, both groups were asked to attend two online health lectures during a 6-month period. These lectures on basic dementia and dementia prevention were given by a certified physician. To present the results of the lessons, an exhibition of the final works was held at the end of all interventions and examinations. The event was also attended by CG participants, and a booth was set up where they could experience digital equipment, thus providing them a place and an opportunity to engage in digital art.

### 2.5 Ethical considerations

This study was conducted with the approval of the Ethics Committee of Tohoku University Graduate School of Medicine (ID: 2024-1-209). Written informed consent was obtained from all participants. The study was registered as a clinical trial in the UMIN-CTR (UMIN000051427).

### 2.6 Statistical analysis

As a baseline assessment, scores on each of the pre-intervention demographic variables and measures of cognitive and psychological function were evaluated using uncorrelated t-tests and chi-square (χ^2^) tests. Although sex is an important demographic variable, sex-specific analyses were not conducted in this study due to the limited sample size associated with the pilot trial design. The sample did not have sufficient statistical power to detect interaction effects or conduct stratified analyses by sex. Therefore, sex was examined only for baseline equivalence between groups. Next, to measure the intervention effects of digital art, an analysis of variance (ANOVA) with linear mixed models was used to examine the main effects of time (pre-post) and group (IG-CG) and the interaction of the two on the dependent variable. In addition, to examine the relationship between changes in drawing skills and scores of cognitive and psychological functions, we divided the groups into two subgroups, one with large changes in drawing skills scores at two time points and the other with small changes. We then conducted an unpaired t-test using the changes in cognitive and psychological functions of the two subgroups as the dependent variables. The significance level for all statistical analyses was set at p < 0.05. However, results with p-values less than 0.10 were also reported as trends to highlight potentially meaningful patterns, while also acknowledging their limited statistical support. Missing values were addressed using multiple imputation methods. The number of pseudo-datasets was set to 10, and the analysis was performed by means of estimates integrating these datasets. SPSS version 27 (IBM, New York, United States) was used for statistical analysis.

## 3 Results

### 3.1 Demographic variables

The means [standard deviations (SD)] of demographic variables for the two groups of participants are shown in Table 2. The chi-square test was performed for sex, current medical history, and past medical history. Meanwhile, independent sample t-tests were applied for age and years of education. No significant differences were found between the IG and the CG for any of these variables, indicating that the characteristics of the groups were homogeneous. In addition, only RAVLT-recognition was significantly higher in the IG for baseline cognitive and psychological function scores (p = 0.028), but no significant differences between the two groups were observed for the other measures.

**TABLE 2:** Demographic variables.

Variable	Intervention group (n = 37)	Control group (n = 35)	p-value
Sex			
Male/Female	18/19	20/15	0.471
Age (SD)	69.30 (2.77)	69.57 (2.67)	0.274
Year of education (SD)	14.38 (1.75)	15.03 (1.69)	0.114
Current medical history			
Yes/No	22/15	18/17	0.636
Past medical history			
Yes/No	24/13	21/14	0.808

The χ^2^ test was used for sex, current medical history and past medical history comparisons and the t-test for age and years of education.

### 3.2 Effects of interventions on cognitive and psychological functions

**FIGURE 3:**
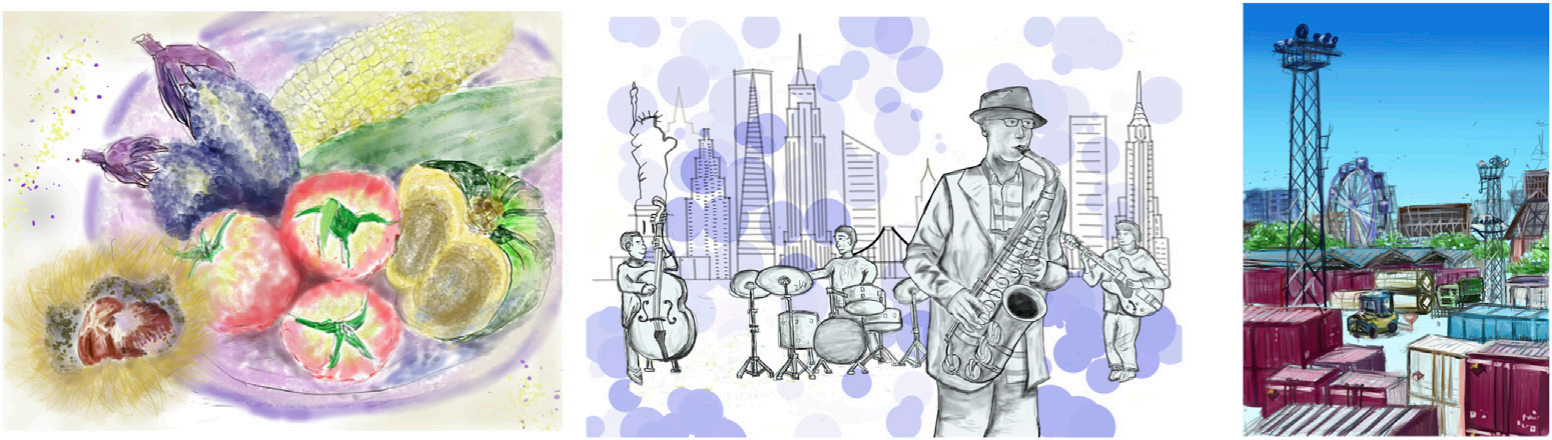
Examples of final artworks created by IG participants.

A linear model-based ANOVA was conducted on the dataset after multiple imputations to examine the main effects of time (pre - post) and group (IG - CG), as well as their interaction, on the dependent variables. The results of the analysis are presented in Table 3. In addition, several examples of final artworks created by IG participants during the sessions are shown in Figure 3.

**TABLE 3:** Means and standard deviations of cognitive and psychological functions and the results of analyses.

Variable	Intervention group (n = 37)				Control group (n = 35)				Group × Time
	Pre		Post		Pre		Post		p-value
	Mean	SD	Mean	SD	Mean	SD	Mean	SD	
FAB	15.95	1.61	16.78	1.40	16.14	1.61	16.20	1.98	0.06^a^
RAVLT-list	46.14	7.44	50.35	9.69	43.77	10.58	48.46	9.06	0.80
RAVLT-immediate	10.16	2.62	11.00	2.80	9.55	2.96	10.48	2.67	0.89
RAVLT-delay	9.65	2.78	10.86	3.27	9.75	3.11	10.52	2.78	0.44
RAVLT-recognition	14.57	0.80	14.81	0.52	14.06	1.11	14.17	1.16	0.66
TMT-A	39.28	10.39	36.72	10.11	39.89	11.27	38.16	10.35	0.74
TMT-B	80.17	29.60	74.31	20.92	77.71	30.16	84.73	53.17	0.20
ΔTMT	40.88	28.63	37.60	30.55	37.81	29.05	46.57	53.44	0.75
CFI	69.57	6.92	70.95	6.30	69.89	8.28	71.34	8.91	0.94
Reverse Stroop Interference (%)	9.42	10.46	11.49	9.19	10.56	9.56	10.59	9.18	0.20
Stroop Interference (%)	14.93	20.83	12.33	12.54	17.28	15.08	16.14	20.24	0.75
D-CAT-1	282.65	61.43	287.49	59.97	286.37	51.00	283.29	59.04	0.49
D-CAT-2	230.27	46.67	227.68	48.78	233.31	40.65	238.40	48.48	0.36
D-CAT-3	174.30	36.15	177.54	38.22	178.74	41.22	188.46	42.49	0.31
Digit span-forward	7.86	2.31	7.76	2.35	7.57	2.12	7.20	2.23	0.51
Digit span-backward	6.68	2.07	6.46	2.06	6.40	1.97	6.60	1.93	0.39
GDS-15-J	2.53	2.45	2.59	2.53	3.23	3.34	3.21	2.78	0.86
SWLS	22.78	5.01	23.54	4.88	21.23	5.89	22.20	5.89	0.75

FAB: the frontal assessment battery; RAVLT: the japanese version of the rey auditory verbal learning test; TMT: the trail making test; ΔTMT: The Trail Making Test part B- The Trail Making Test part A; CFI: cognitive flexibility inventory; D-CAT: the digit cancellation test; GDS-15-J: The shortened version of the Japanese version of the Geriatric Depression Scale 15; SWLS: the satisfaction with life scale.

^a^ p < 0.10.

For the FAB, while no significant interaction between time and group was found, a significant trend was observed [t = 1.864, SE = 0.419, 95% confidence interval CI (−0.040, 1.602), p = 0.062]. Post hoc tests with Bonferroni correction indicated that in the IG, post-intervention scores were significantly higher than were pre-intervention scores [t = −3.170, SE = 0.264, 95% CI (−1.356, −0.320), p = 0.002], whereas in the CG, no significant difference was found between pre- and post-intervention scores [t = −0.174, SE = 0.328, 95% CI (−0.700, 0.585), p = 0.862]. In addition, no significant differences were observed between the IG and CG at either time point [pre: t = 0.518, SE = 0.380, 95% CI (−0.549, 0.942), p = 0.605; post: t = −1.451, SE = 0.402, 95% CI (−1.372, 0.212), p = 0.147].

Regarding the RAVLT, significant main effects of time were observed for the list learning(t = −3.470, SE = 1.215, 95% CI [-6.598, −1.835], p = 0.001), immediate recall [t = −2.056, SE = 0.408, 95% CI (−1.637, −0.039), p = 0.040], and delayed recall [t = −3.173, SE = 0.383, 95% CI (−1.967, −0.465), p = 0.002] components, with significantly higher post-intervention scores observed in both the IG and CG. A significant main effect of group was found for RAVLT-recognition [t = −2.882, SE = 0.224, 95% CI (−1.084, −0.206), p = 0.004], with the IG showing significantly higher scores than the CG at both pre- and post-intervention time points. No statistically significant results were observed for other measures of cognitive function or psychological function, such as GDS-15-J and SWLS.

### 3.3 The relationship between drawing skill and cognitive and psychological functions

The relationship between drawing skill—an indicator of learning outcomes resulting from the intervention—and cognitive and psychological functions was examined. Specifically, the association between changes in drawing ability (post–pre scores on the drawing skill test) and changes in cognitive and psychological functions (pre–post scores on each measure) were analyzed within the IG.

**FIGURE 4:**
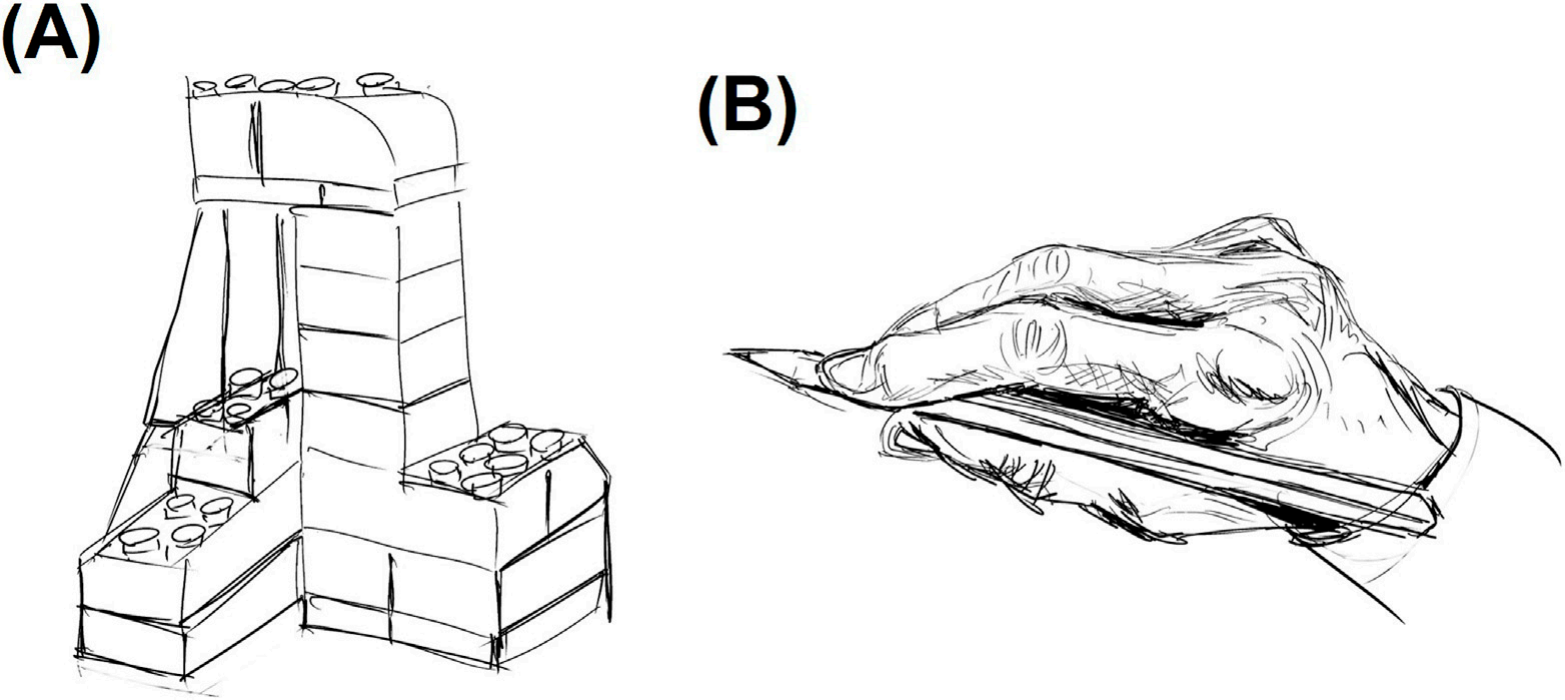
Outputs of drawing skills tasks: **(A)** Task A (block); **(B)** Task B (hand with pencil).

The mean (SD) scores on the drawing skill test were 6.08 (2.19) and 8.24 (3.10) at pre-and post-intervention across the IG. As the median change from pre to post was 3 points, participants were categorized into a “high improvement group” (≥3-point improvement) and a “low improvement group” (<3-point improvement) consisting of 19 and 18 participants, respectively. Descriptive statistics for each group’s drawing ability test scores are presented in Table 4. In addition, several outputs of drawing skills tasks conducted on actual participants are shown in Figure 4. Independent samples t-tests were conducted with the group as the independent variable and the change scores in each cognitive and psychological function as dependent variables. A significant group difference was observed only for the RAVLT-list scores, with the low improvement group showing significantly greater improvement [t = 2.156, SE = 2.012, 95% CI (0.395, 8.284), p = 0.031]. No significant differences were observed for the other measures and no other significant main effects or interactions were observed for the remaining outcome measures.

**TABLE 4:** Means and standard deviations of drawing skill test scores in each group.

Time	High - group (n=19)		Low - group (n=18)	
	Mean	SD	Mean	SD
Pre	6.11	2.72	6.06	1.57
Post	10.16	2.33	6.22	2.52
Post - Pre	4.05	1.23	0.17	1.99

## 4 Discussion

This study investigated the effects of a 20-session digital visual art learning intervention on cognitive and psychological functions in healthy older adults aged 65 to 74 with no prior experience in art. Although statistical significance was not reached, the IG demonstrated a trend toward improvement on the FAB, a measure of frontal lobe function. In contrast, no significant effects were observed on other cognitive or psychological indicators, suggesting that engagement in digital art could potentially enhance cognitive functions related to the frontal lobe, such as executive function, in older adults.

A review of previous studies revealed that while cognitive benefits of visual arts interventions have been reported for older adults with cognitive decline, clear cognitive improvements are less frequently observed in healthy older adults without cognitive impairment (Masika et al., 2020). One possible explanation is that individuals with cognitive decline may exhibit greater cognitive plasticity than do those without cognitive decline (Rosebud and Knopman, 2013), highlighting the challenge of identifying effective activities for cognitively healthy populations (Masika et al., 2020). However, an intervention study by Brown et al. (2021), which targeted healthy older adults without cognitive impairment, reported significant improvements in executive function and cognitive flexibility following a conceptual art intervention, which included elements of visual arts. This finding is partially consistent with the results of the present study. Drawing engages a variety of cognitive functions, including attention, decision-making, motor control, visual information processing, and working memory (Katz et al., 2021). Notably, creative drawing processes activate the executive control network in the prefrontal cortex (Bolwerk et al., 2014; De Pisapia et al., 2016). Associations with similar frontoparietal networks have also been reported (Raimo et al., 2021), suggesting that such creative engagement may induce structural changes in regions such as the medial frontal gyrus, a part of the frontal lobe (Chamberlain et al., 2014). Therefore, sustained engagement in digital art—an inherently creative activity—may contribute to observable improvements in executive functions, particularly those related to frontal lobe activity. However, it is noteworthy that, in this study, the FAB exhibited a trend toward improvement in executive function, while other tests that also measure executive function, such as the TMT and the Stroop task, did not show clear changes. This result may be because of differences in the domain of executive function that each test measures. The FAB was developed as a comprehensive measure of executive function (Dubois et al., 2000). The TMT, on the other hand, focuses primarily on cognitive flexibility, while the Stroop task focuses on specific executive functions such as inhibitory control. Therefore, aspects of executive function that revealed improvements on the FAB may have not matched those measured on the TMT or the Stroop task. In addition, since mental fatigue affects cognitive performance (Van der Linden et al., 2003), the order in which the tests were administered may have played a significant role in the results. Specifically, participants may have felt more mental fatigue during the TMT and the Stroop task, which were administered in the middle-to-late stages of the test, than during the FAB, which was administered at the beginning. This may have affected the results. In the RAVLT, although a significant main effect of time was observed, both the IG and CG showed increased scores, suggesting that the observed improvements were likely due to practice effects. No clear improvements were found in the other cognitive indicators. Masika et al. (2020) contend that key features of effective visual art interventions involve components requiring a high level of cognitive engagement including art analysis/cognitive evaluation and tasks that stimulate creativity during art processing. Although the current program included a variety of tasks designed to stimulate creativity, the components involving art analysis, cognitive evaluation, and reflection were limited to brief write-ups at the end of each session. Ample time for deeper introspection or focused verbalization was not incorporated, resulting in potentially insufficient cognitive engagement to improve functions other than executive function.

No effects of the intervention were observed on psychological functions, as measured by the scores on depression symptoms (GDS-15-J) and life satisfaction (SWLS). Previous studies on visual arts interventions for older adults with diagnosed depression or depressive tendencies have reported improvements in depressive symptoms (Choi and Jeon, 2013; Ciasca et al., 2018; Im and Lee, 2014; Hsu et al., 2017; Kang et al., 2010) and self-esteem (Ching-Teng et al., 2019). These interventions were typically carried out by professional art therapists under therapeutic settings, dedicating considerable time to the verbal interpretation and discussion of artworks. According to Dunphy et al. (2019), the emotional and internal mechanisms of such interventions involve the creation of art, providing a valuable distance for the externalization of internal subjective experiences and enabling visual communication. In addition, the completion of the artwork contributes to the strengthening of agency. The intervention in this study was intended for healthy older adults and was, therefore, more of a learning experience rather than a therapeutic intervention by a professional art therapist. No active approach to the psychological aspects of the participants was adopted by researchers and art demonstrators, as the program content predominantly focused on teaching and exploring digital art techniques.

A study by Kim (2013), that also targeted healthy older adults, revealed certain psychological improvements, such as reductions in negative emotions and anxiety and increases in self-esteem. However, the intervention included significant time spent on group discussions (15–20 min of a 60–75 min session). In contrast, the intervention in this study allotted only approximately 3–5 min at the end of each session to complete a brief reflection sheet and share any impressions. The content of the reflection sheet included determining the participants’ understanding of the day’s lesson, their emotions during the session, and optional, non-mandatory free-text feedback on the session. Based on these observations, the lack of psychological improvements in the present study could be attributed to several factors: the relatively good mental health of participants prior to the intervention, limited involvement by intervenors, and time allocated for externalizing and verbally processing participants' internal subjective experiences through art. The time allocated for deep introspection or discussion among participants was insufficient.

No statistically significant relationship was found between changes in drawing skills resulting from the digital visual art intervention and changes in cognitive or psychological functions. Previous research has revealed that training in visual arts can lead to changes in neural processing within brain regions that mediate the integration of perception and action (Schlegel et al., 2015). In addition, it has been suggested that drawing expertise may not stem from improved perceptual abilities but from enhanced capacity to transform perceived information into creative actions (Perdreau and Cavanagh, 2011). From this perspective, while artistic drawing itself may serve as a stimulus contributing to cognitive function, the process of acquiring drawing expertise emphasizes integrating perceived information with motor control and coordination between eye and hand movements—functions supported by complex brain networks. Therefore, improvements in drawing expertise may be more closely reflected in changes in brain network connectivity than in improvements in specific subdomains of cognitive function as measured in the present study. Furthermore, the drawing ability assessment employed in this study was originally developed as a general measure of traditional drawing skills (Chamberlain et al., 2014). However, acquiring digital visual art skills involves not only basic artistic abilities but also the effective use of digital software and tools. The skills participants developed through the program included effective use of pen functions, layer management, selection tools, and diverse coloring techniques—elements beyond conventional drawing activities. Thus, the learning outcomes of the program may not have been fully captured by a drawing skill test focused solely on traditional drawing ability. Regarding psychological functions, as noted by Dunphy et al. (2019), the internal and subjective world of participants plays a critical role. Therefore, participants’ subjective sense of achievement—such as feeling that they had “improved”—may be more strongly associated with psychological effects than with objective evaluations of drawing ability. In light of these considerations, future research should revisit the evaluation indicators used to assess the relationship between drawing skill and cognitive/psychological functions. Refining both the task design and evaluation methods to better reflect the specific learning content of the program will be an important challenge.

This study has several limitations. First, its results only exhibited significant trends rather than a clear significant difference; therefore, they should be interpreted cautiously. The results of this study demonstrated trends toward improvement rather than statistically significant differences. Several factors may have contributed to this outcome, including limitations in sample size, insufficient control of confounding variables such as participants’ social and leisure activities, potential shortcomings in the newly developed intervention program, and possible mismatches between the selected outcome measures and the actual effects of the intervention. As a pioneering attempt, this study is positioned as a pilot investigation, and the findings provide valuable insights for future research. Moving forward, it will be essential to address these limitations by expanding the sample size, refining the research design. Second, the study did not incorporate measures related to social interaction. Masika et al. (2020), Masika et al. (2022) contended that visual art exerts its effects on cognitive and psychological functioning through the three mechanisms of physiological, cognitive, and social stimulations. In the present study, each group consisted of fixed members, and the long-term nature of the program appeared to foster the formation of small-scale communities among participants. Social interaction has been shown to positively influence both cognitive function (Kelly et al., 2017) and psychological wellbeing (Nyqvist et al., 2013) among older adults. Future studies should include indicators of social interaction and analyze their associations with cognitive and psychological outcomes to allow for a more comprehensive and ecologically valid understanding of the effects of digital art learning. Third, while this study employed quantitative methods to analyze the data, quantitative analysis alone could not capture the subjective and individualized experiences of participants throughout the digital art learning process. Previous research has utilized qualitative methods to explore participants’ inner experiences in greater depth (Cantu and Fleuriet, 2017; MacLeod et al., 2016; Rose and Lonsdale, 2016; Stickley et al., 2016). In addition, some studies have adopted mixed-methods approaches combining both quantitative and qualitative data (Camic et al., 2014; Phinney et al., 2014). These studies have provided rich insights into participants’ emotional states during the creative process, their renewed appreciation of the world and its beauty (Cantu and Fleuriet, 2017), personal growth (e.g., acquiring new abilities through the creation of art), and a sense of meaning and fulfillment (e.g., personal expression, perceived significance, and satisfaction) (MacLeod et al., 2016)—aspects that go beyond what can be measured through quantitative indicators alone. Therefore, future research should incorporate qualitative approaches in addition to quantitative methods to further elucidate the multifaceted effects of digital art learning on older adults. Moreover, the program content and instructional methods should be continuously updated to optimize its effectiveness in maintaining and enhancing cognitive and psychological functioning. Therefore, future studies should address the identified limitations and advance the development of more refined and adaptable digital art programs.

## 5 Conclusion

This study details one of the first attempts to examine the effects of digital visual art learning on cognitive and psychological functions in healthy older adults. Its results suggest that digital visual art learning tends to improve frontal lobe functions, mainly executive functions. The findings of this study highlight the potential utility of digital visual arts learning as an innovative, non-pharmacological approach to support cognitive function in older adults and may promote healthy longevity in the community. Future studies should develop effective and adaptive digital art learning programs for older adults by increasing the sample size, while examining indices of social interaction and adopting a more individualized approach using qualitative methods as well.

## Data Availability

The raw data supporting the conclusions of this article will be made available by the authors, without undue reservation.

## References

[B1] Beauchet O. Bastien T. Mittelman M. Hayashi Y. Ho A. H. Y. (2020). Participatory art-based activity, community-dwelling older adults and changes in health condition: results from a pre–post intervention, single-arm, prospective and longitudinal study. Maturitas 134, 8–14. 10.1016/j.maturitas.2020.01.006 32143777

[B2] Bolwerk A. Mack-Andrick J. Lang F. R. Dörfler A. Maihöfner C. (2014). How art changes your brain: differential effects of visual art production and cognitive art evaluation on functional brain connectivity. PloS One 9, e101035. 10.1371/journal.pone.0101035 24983951 PMC4077746

[B3] Bone J. K. Fancourt D. Sonke J. K. Bu F. (2024). Participatory and receptive arts engagement in older adults: associations with cognition over a seven-year period. Creat. Res. J. 36 (3), 436–450. 10.1080/10400419.2023.2247241 39140023 PMC11318508

[B4] Bowie C. R. Harvey P. D. (2006). Administration and interpretation of the trail making test. Nat. Protoc. 1 (5), 2277–2281. 10.1038/nprot.2006.390 17406468

[B5] Bowling A. (2007). Aspirations for older age in the 21st century: what is successful ageing? Int. J. Aging. Hum. Dev. 64, 263–297. 10.2190/L0K1-87W4-9R01-7127 17503689

[B6] Brown C. J. Chirino A. F. C. Cortez C. M. Gearhart C. Urizar G. G. (2021). Conceptual art for the aging brain: piloting an art-based cognitive health intervention. Adapt. Aging. 45, 39–69. 10.1080/01924788.2020.1719584

[B7] Camic P. M. Tischler V. Pearman C. H. (2014). Viewing and making art together: a multi-session art-gallery-based intervention for people with dementia and their carers. Aging Ment. Health 18 (2), 161–168. 10.1080/13607863.2013.818101 23869748

[B8] Camurri A. Seminerio E. Morganti W. Canepa C. Ferrari N. Ghisio S. (2024). Development and validation of an art-inspired multimodal interactive technology system for a multi-component intervention for older people: a pilot study. Front. Comput. Sci. 5, 1290589. 10.3389/fcomp.2023.1290589

[B9] Cantu A. G. Fleuriet K. J. (2017). “Making the ordinary more extraordinary”: exploring creativity as a health promotion practice among older adults in a community-based professionally taught arts program. J. Holist. Nurs. 36 (2), 123–133. 10.1177/0898010117697863 29172944

[B10] Cetinkaya F. Duru Asiret G. Direk F. Özkanli N. N. (2019). The effect of ceramic painting on the life satisfaction and cognitive status of older adults residing in a nursing home. Top. Geriatr. Rehabil. 35 (2), 108–112. 10.1097/TGR.0000000000000208

[B11] Chamberlain R. McManus I. C. Brunswick N. Rankin Q. Riley H. Kanai R. (2014). Drawing on the right side of the brain: a voxel-based morphometry analysis of observational drawing. Neuroimage 96, 167–173. 10.1016/j.neuroimage.2014.03.062 24691200

[B12] Ching-Teng Y. Ya-Ping Y. Yu-Chia C. (2019). Positive effects of art therapy on depression and self-esteem of older adults in nursing homes. Soc. Work Health Care 58 (3), 324–338. 10.1080/00981389.2018.1564108 30628552

[B13] Choe S. (2014). An exploration of the qualities and features of art apps for art therapy. Arts. Psychother. 41, 145–154. 10.1016/j.aip.2014.01.002

[B14] Choi Y. H. Jeon E. Y. (2013). Effects of art therapy on cognition, depression, and quality of life in elderly. J. Korean Acad. Comm. Health Nurs. 24, 323–331. 10.12799/jkachn.2013.24.3.323

[B15] Ciasca E. C. Ferreira R. C. Santana C. L. A. Forlenza O. V. Dos Santos G. D. Brum P. S. (2018). Art therapy as an adjuvant treatment for depression in elderly women: a randomized controlled trial. Rev. Bras. Psiquiatr. 40 (3), 256–263. 10.1590/1516-4446-2017-2250 29412335 PMC6899401

[B16] Darewych O. H. Carlton N. R. Farrugie K. W. (2015). Digital technology use in art therapy with adults with developmental disabilities. J. Dev. Disabil. 21 (2), 95–102.

[B17] De Pisapia N. Bacci F. Parrott D. Melcher D. (2016). Brain networks for visual creativity: a functional connectivity study of planning a visual artwork. Sci. Rep. 6 (1), 39185. 10.1038/srep39185 27991592 PMC5171814

[B18] de Sousa Magalhães S. Malloy-Diniz L. F. Hamdan A. C. (2012). Validity convergent and reliability test-retest of the Rey Auditory Verbal Learning Test. Clin. Neuropsychiatry 9 (3), 129–137.

[B19] Dennis J. P. Vander Wal J. S. (2010). The cognitive flexibility inventory: instrument development and estimates of reliability and validity. Cogn. Ther. Res. 34, 241–253. 10.1007/s10608-009-9276-4

[B20] Di Giacomo D. Ranieri J. D’Amico M. Guerra F. Passafiume D. (2019). Psychological barriers to digital living in older adults: computer anxiety as predictive mechanism for technophobia. Behav. Sci. 9 (9), 96. 10.3390/bs9090096 31514364 PMC6770433

[B21] Du Y. Peng R. Wan X. Zhang C. Chang J. Guo Y. (2025). “Digital creative art interventions on health promotion among older adults: a scoping review. J. Clin. Nurs. 17787. 10.1111/jocn.17787 40369710

[B22] Dubois B. Slachevsky A. Litvan I. Pillon B. (2000). The FAB: a frontal assessment battery at bedside. Neurology 55, 1621–1626. 10.1212/WNL.55.11.1621 11113214

[B23] Dunphy K. Baker F. A. Dumaresq E. Carroll-Haskins K. Eickholt J. Ercole M. (2019). Creative arts interventions to address depression in older adults: a systematic review of outcomes, processes, and mechanisms. Front. Psychol. 9, 2655. 10.3389/fpsyg.2018.02655 30671000 PMC6331422

[B24] Elliott R. (2003). Executive functions and their disorders. Br. Med. Bull. 65, 49–59. 10.1093/BMB/65.1.49 12697616

[B25] Groot B. de Kock L. Liu Y. Dedding C. Schrijver J. Teunissen T. (2021). The value of active arts engagement on health and well-being of older adults: a nation-wide participatory study. Int. J. Environ. Res. Public Health. 18 (15), 8222. 10.3390/ijerph18158222 34360519 PMC8345976

[B26] Guo J. Huang X. Dou L. Yan M. Shen T. Tang W. (2022). Aging and aging-related diseases: from molecular mechanisms to interventions and treatments. Signal Transduct. Target. Ther. 7, 391. 10.1038/s41392-022-01251-0 36522308 PMC9755275

[B27] Hakoda Y. Sasaki M. (1990). Group version of the Stroop and reverse-Stroop test (in Japanese). Jpn. J. Educ. Psychol. 38 (4), 389–394. 10.5926/jjep1953.38.4_389 8355429

[B28] Hakoda Y. Watanabe M. (2005). New stroop Test II. Fukuoka: toyo physical.

[B29] Hardy M. Oprescu F. Millear P. Summers M. (2017). Baby boomers engagement as traditional university students: benefits and costs. Int. J. Lifelong Educ. 36 (6), 730–744. 10.1080/02601370.2017.1382015

[B30] Hatta T. Yoshizaki K. Ito Y. Mase M. Kabasawa H. (2012). Reliability and validity of the digit cancellation test, a brief screen of attention. Psychologia 55 (4), 246–256. 10.2117/psysoc.2012.246

[B31] Hatta T. Ito Y. Yoshizaki K. (2023). D-CAT: the digit cancellation test, A brief screen of attention. 4th ed. Osaka: FIS, Inc.

[B32] Hattori H. Hattori C. Hokao C. Mizushima K. Mase T. (2011). Controlled study on the cognitive and psychological effect of coloring and drawing in mild alzheimer's disease patients. Geriatr. Gerontol. Int. 11, 431–437. 10.1111/j.1447-0594.2011.00698.x 21518170

[B33] Hsu T. J. Tsai H. T. Hwang A. C. Chen L. Y. Chen L. K. (2017). Predictors of non-pharmacological intervention effect on cognitive function and behavioral and psychological symptoms of older people with dementia. Geriatr. Gerontol. Int. 17, 28–35. 10.1111/ggi.13037 28436192

[B34] Idowu M. Szameitat A. (2023). Executive function abilities in cognitively healthy young and older adults—A cross-sectional study. Front. Aging Neurosci. 15, 976915. 10.3389/fnagi.2023.976915 36845657 PMC9945216

[B35] Im M. L. Lee J. I. (2014). Effects of art and music therapy on depression and cognitive function of the elderly. Technol. Health Care 22, 453–458. 10.3233/THC-140803 24704654

[B36] Japan Society for Higher Brain Function. (2019). Trail making Test, Japanese edition (TMT-J). Tokyo: Shinkoh-Igaku shuppan.

[B37] Kaimal G. Carroll-Haskins K. Berberian M. Dougherty A. Carlton N. Ramakrishnan A. (2019). Virtual reality in art therapy: a pilot qualitative study of the novel medium and implications for practice. Art. Ther. 37 (1), 16–24. 10.1080/07421656.2019.1659662

[B38] Kang H. Bae Y. Kim E. Lee K. Chae M. Ju R. (2010). An integrated dementia intervention for Korean older adults. J. Psychosoc. Nurs. Ment. Health Serv. 48, 42–50. 10.3928/02793695-20100930-01 21053789

[B39] Katz J. S. Forloines M. R. Strassberg L. R. Bondy B. (2021). Observational drawing in the brain: a longitudinal exploratory fMRI study. Neuropsychologia 160, 107960. 10.1016/j.neuropsychologia.2021.107960 34274380

[B40] Kelly M. E. Duff H. Kelly S. McHugh Power J. E. Brennan S. Lawlor B. A. (2017). The impact of social activities, social networks, social support and social relationships on the cognitive functioning of healthy older adults: a systematic review. Syst. Rev. 6, 259–18. 10.1186/s13643-017-0632-2 29258596 PMC5735742

[B41] Kim S. K. (2013). A randomized, controlled study of the effects of art therapy on older Korean-Americans’ healthy aging. Arts Psychother. 40, 158–164. 10.1016/j.aip.2012.11.002

[B42] Kim J. Chung Y. J. (2023). A case study of group art therapy using digital media for adolescents with intellectual disabilities. Front. Psychiatry. 14, 1172079. 10.3389/fpsyt.2023.1172079 37200905 PMC10187545

[B43] Kim H. N. Freddolino P. P. Greenhow C. (2023). Older adults’ technology anxiety as a barrier to digital inclusion: a scoping review. Educ. Gerontol. 49 (12), 1021–1038. 10.1080/03601277.2023.2202080

[B44] Kongkasuwan R. Voraakhom K. Pisolayabutra P. Maneechai P. Boonin J. Kuptniratsaikul V. (2016). Creative art therapy to enhance rehabilitation for stroke patients: a randomized controlled trial. Clin. Rehabil. 30 (10), 1016–1023. 10.1177/0269215515607072 26396163

[B45] Leanos S. Kürüm E. Strickland-Hughes C. M. Ditta A. S. Nguyen G. Felix M. (2023). The impact of learning multiple real-world skills on cognitive abilities and functional independence in healthy older adults. J. Gerontol. B Psychol. Sci. Soc. Sci. 78 (8), 1305–1317. 10.1093/geronb/gbad053 37171401 PMC10394988

[B46] Lee R. Wong J. Shoon W. L. Gandhi M. Lei F. Eh K. (2019). Art therapy for the prevention of cognitive decline. Arts Psychother. 64, 20–25. 10.1016/j.aip.2018.12.003

[B47] MacLeod A. Skinner M. W. Wilkinson F. Reid H. (2016). Connecting socially isolated older rural adults with older volunteers through expressive arts. Can. J. Aging./La Rev. Can. Du. Vieil. 35 (1), 14–27. 10.1017/S071498081500063X 26934547

[B48] Mahendran R. Gandhi M. Moorakonda R. B. Wong J. Kanchi M. M. Fam J. (2018). Art therapy is associated with sustained improvement in cognitive function in the elderly with mild neurocognitive disorder: findings from a pilot randomized controlled trial for art therapy and music reminiscence activity *versus* usual care. Trials 19 (1), 615–10. 10.1186/s13063-018-2988-6 30413216 PMC6230219

[B49] Masika G. M. Yu D. S. Li P. W. (2020). Visual art therapy as a treatment option for cognitive decline among older adults. A systematic review and meta‐analysis. J. Adv. Nurs. 76 (8), 1892–1910. 10.1111/jan.14362 32201968

[B50] Masika G. M. Yu D. S. F. Li P. W. C. Lee D. T. F. Nyundo A. (2022). Visual art therapy and cognition: effects on people with mild cognitive impairment and low education level. J. Gerontol. B Psychol. Sci. Soc. Sci. 77, 1051–1062. 10.1093/geronb/gbab168 34536278

[B51] McQuade L. O’Sullivan R. (2023). Examining arts and creativity in later life and its impact on older people’s health and wellbeing: a systematic review of the evidence. Perspect. Public Health 144 (6), 344–353. 10.1177/17579139231157533 36905227

[B52] Moradi E. Hallikainen I. Hänninen T. Tohka J. Alzheimer's Disease Neuroimaging Initiative (2017). Rey’s auditory verbal learning test scores can be predicted from whole brain MRI in Alzheimer’s disease. Neuroimage Clin. 13, 415–427. 10.1016/j.nicl.2016.12.011 28116234 PMC5233798

[B53] Nakaaki S. Murata Y. Sato J. Shinagawa Y. Matsui T. Tatsumi H. (2007). Reliability and validity of the Japanese version of the frontal assessment battery in patients with the frontal variant of frontotemporal dementia. Psychiatry Clin. Neurosci. 61 (1), 78–83. 10.1111/j.1440-1819.2007.01614.x 17239043

[B54] Nihon Bunka Kagakusha (2018). WAIS-IV Japanese version manual. Nihon Bunka Kagakusha.

[B55] Noice H. Noice T. Staines G. (2004). A short-term intervention to enhance cognitive and affective functioning in older adults. J. Aging Health 16 (4), 562–585. 10.1177/0898264304265819 15271270

[B56] Noice T. Noice H. Kramer A. F. (2014). Participatory arts for older adults: a review of benefits and challenges. Gerontologist 54 (5), 741–753. 10.1093/geront/gnt138 24336875 PMC4229893

[B57] Nyqvist F. Forsman A. K. Giuntoli G. Cattan M. (2013). Social capital as a resource for mental well-being in older people: a systematic review. Aging Ment. Health 17 (4), 394–410. 10.1080/13607863.2012.742490 23186534

[B58] Oishi S. (2009). The science of happiness: what we know from psychology. Tokyo: Shinyosha.

[B59] Özdemir D. (2022). A conceptual framework on the relationship of digital technology and art. Int. J. Soc. Educ. Sci. 4 (1), 121–134. 10.46328/ijonses.313

[B60] Perdreau F. Cavanagh P. (2011). Do artists see their retinas? Front. Hum. Neurosci. 5, 171. 10.3389/fnhum.2011.00171 22232584 PMC3248676

[B61] Phinney A. Moody E. M. Small J. A. (2014). The effect of a community-engaged arts program on older adults’ well-being. Can. J. Aging./La Rev. Can. Du. Vieil. 33 (3), 336–345. 10.1017/S071498081400018X 25110936

[B62] Pongan E. Tillmann B. Leveque Y. Trombert B. Getenet J. C. Auguste N. (2017). Can musical or painting interventions improve chronic pain, mood, quality of life, and cognition in patients with mild Alzheimer's disease? Evidence from a randomized controlled trial. J. Alzheimers Dis. 60 (2), 663–677. 10.3233/JAD-170410 28922159

[B63] Raimo S. Santangelo G. Trojano L. (2021). The neural bases of drawing. A meta-analysis and a systematic literature review of neurofunctional studies in healthy individuals. Neuropsychol. Rev. 31, 689–702. 10.1007/s11065-021-09494-4 33728526 PMC8593049

[B64] Rey A. (1964). L’ examen clinique en psychogie [The Clinical Examination in Psychology]. Paris: Presses Universitaires de France.

[B65] Rincon-Gómez A. M. Garcia-Flórez J. A. Suescum M. F. Sierra L. F. Maya J. (2016). Assessing line, proportion, precision and perspective in traditional drawing method and digital pen-based technology for familiarized subjects. ICERI2016 Proc. IATED. 7714-7723. 10.21125/iceri.2016.0768

[B66] Rose E. Lonsdale S. (2016). Painting place: re-imagining landscapes for older people's subjective wellbeing. Health Place 40, 58–65. 10.1016/j.healthplace.2016.05.002 27179603

[B67] Rosebud R. Knopman D. S. (2013). Classification and epidemiology of MCI. Clin. Geriatr. Med. 29 (4), 1–19. 10.1016/j.cger.2013.07.003 24094295 PMC3821397

[B68] Sánchez-Cubillo I. Periáñez J. Adrover-Roig D. Rodríguez-Sánchez J. Ríos-Lago M. Tirapu J. (2009). Construct validity of the Trail making test: role of task-switching, working memory, inhibition/interference control, and visuomotor abilities. J. Int. Neuropsychol. Soc. 15, 438–450. 10.1017/S1355617709090626 19402930

[B69] Schlegel A. Alexander P. Fogelson S. V. Li X. Lu Z. Kohler P. J. (2015). The artist emerges: visual art learning alters neural structure and function. NeuroImage 105, 440–451. 10.1016/j.neuroimage.2014.11.014 25463452

[B70] Schmidt M. (1996). Rey auditory verbal learning test: a handbook. Los Angeles: Western Psychological Services.

[B71] Schoultz M. Öhman J. Quennerstedt M. (2020). A review of research on the relationship between learning and health for older adults. Int. J. Lifelong Educ. 39 (5-6), 528–544. 10.1080/02601370.2020.1819905

[B72] Srivastava A. (2019). Digital art: a revolutionary form of art and visual communication. Int. J. Res. -GRANTHAALAYAH 7 (11), 83–88. 10.29121/granthaalayah.v7.i11.2019.3705

[B73] Stickley T. Hui A. Souter G. Mills D. (2016). A community arts programme for older people: an evaluation. Health Soc. Incl. 20 (1), 22–28. 10.1108/MHSI-07-2015-0027

[B74] Sugishita K. Sugishita M. Hemmi I. Asada T. Tanigawa T. (2016). A validity and reliability study of the Japanese version of the geriatric depression scale 15 (GDS-15-J). Clin. Geronto 40 (4), 233–240. 10.1080/07317115.2016.1199452 28452641

[B75] Sugishita M. Asada T. Sugishita K. (2017). Japanese version of the geriatric depression scale 15 (GDS-15-J). Tokyo: Shinkoh Igaku Shuppnan. 10.1080/07317115.2016.1199452 28452641

[B76] Tokuyoshi Y. Iwasaki S. (2012). “Development and validation of cognitive flexibility inventory -Japanese (in Japanese),” in Proceedings of the 76th annual convention of the Japanese psychological association. 672.

[B77] Tymoszuk U. Perkins R. Spiro N. Williamon A. Fancourt D. (2020). Longitudinal associations between short-term, repeated, and sustained arts engagement and well-being outcomes in older adults. J. Gerontol. B Psychol. Sci. Soc. Sci. 75 (7), 1609–1619. 10.1093/geronb/gbz085 31287550 PMC7424284

[B78] United Nations Department of Economic and Social Affairs, Population Division (2022). World population prospects 2022: summary of results. Available online at: https://www.un.org/development/desa/pd/sites/www.un.org.development.desa.pd/files/wpp2022_summary_of_results.pdf (Accessed March 10, 2024).

[B79] Uno A. Browne R. Shinada T. Takahashi M. Soga K. Du Y. (2025). Digital visual art learning for cognitive and psychological functioning among healthy older adults: a randomized controlled trial protocol. Preprint (version 2). Res. Square. 10.21203/rs.3.rs-4836281/v2

[B80] Van der Linden D. Frese M. Meijman T. F. (2003). Mental fatigue and the control of cognitive processes: effects on perseveration and planning. Acta Psychol. 113, 45–65. 10.1016/S0001-6918(02)00150-6 12679043

[B81] Wagner S. Helmreich I. Dahmen N. Lieb K. Tadić A. (2011). Reliability of three alternate forms of the trail making tests A and B. Arch. Clin. Neuropsychol. 26 (4), 314–321. 10.1093/arclin/acr024 21576092

[B82] Wakamatsu N. Anamizu S. Kato M. (2003). Rey auditory verbal learning test (RAVLT). Nihon Rinsho. Spec. issue, 279–284.

[B83] Wechsler D. (1987). WMS-R: Wechsler memory scale–revised: manual. San Antonio, TX: Psychological Corporation.

[B84] World Health Organization. (2015). World report on ageing and health. Available online at: https://www.who.int/publications/i/item/9789241565042 (Accessed March 9, 2024).

[B85] Zhao J. Li H. Lin R. Wei Y. Yang A. (2018). Effects of creative expression therapy for older adults with mild cognitive impairment at risk of alzheimer's disease: a randomized controlled clinical trial. Clin. Interv. Aging 13, 1313–1320. 10.2147/CIA.S161861 30087557 PMC6063252

